# A grape seed extract maternal dietary supplementation improves egg quality and reduces ovarian steroidogenesis without affecting fertility parameters in reproductive hens

**DOI:** 10.1371/journal.pone.0233169

**Published:** 2020-05-14

**Authors:** Alix Barbe, Namya Mellouk, Christelle Ramé, Jérémy Grandhaye, Karine Anger, Marine Chahnamian, Patrice Ganier, Aurélien Brionne, Antonella Riva, Pascal Froment, Joëlle Dupont

**Affiliations:** 1 INRAE UMR85 Physiologie de la Reproduction et des Comportements, Nouzilly, France; 2 CNRS UMR7247 Physiologie de la Reproduction et des Comportements, Nouzilly, France; 3 Université François Rabelais de Tours, Tours, France; 4 IFCE Nouzilly, Nouzilly, France; 5 INRAE - Unité Expérimentale du Pôle d’Expérimentation Avicole de Tours UEPEAT, 1295, Nouzilly, Nouzilly, France; 6 INRAE, UMR0083 Biologie des Oiseaux et Aviculture, Nouzilly, France; 7 INDENA, Tours, France; University of Illinois, UNITED STATES

## Abstract

In broiler hens, the genetic selection increased susceptibility to metabolic disorders and reproductive dysfunctions. In human ovarian cells, grape seed extracts (GSE) improved steroid production. Here, we investigated the effects of a GSE dietary supplementation on egg production and quality, fertility parameters, Reactive Oxygen Species (ROS) and steroid content in yolk egg associated to plasma adipokines in broiler hens. For this, we designed two *in vivo* experiments, the first one included three groups of hens: A (control), B and C (supplemented with GSE at 0.5% and 1% of the total diet composition, respectively, since week 4), and the second one used two groups of hens: A (control) and D (supplemented with GSE at 1% of the total diet composition since hatching). We assessed the egg production from 23^th^ to 40^th^ weeks and quality at 33^th^ week. After artificial inseminations, the fertility parameters were calculated. In egg yolk, Reactive Oxygen Species (ROS) level and steroid production were evaluated by Ros-Glo H202 and ELISA assay, respectively. Expression of steroidogenic enzymes and adipokines and their receptors was determined by RT-qPCR in ovarian cells and plasma adipokines (RARRES2, ADIPOQ and NAMPT) were evaluated by specific ELISA assays. The fertility parameters and egg production were unaffected by GSE supplementation whatever the experiment (exp.). However, the rate of double-yolk eggs decreased for all GSE supplemented groups (exp. 1 *P* <0.01, exp.2, *P*<0.02). In exp.1, C group eggs were bigger and larger (*P*<0.0001) and the shell elasticity was higher for both B and C (*P*<0.0003) as compared to control. In the egg yolk, GSE supplementation in both exp. reduced ROS content and steroidogenesis consistent with a decrease in P450 aromatase and StAR mRNA expression and basal *in vitro* progesterone secretion in granulosa cells (*P*<0.001). Interestingly, in both exp. RARRES2 plasma levels were positively correlated while ADIPOQ and NAMPT plasma levels were negatively correlated, with steroids and ROS in yolk (*P*<0.0001). Taken together, maternal dietary GSE supplementation did not affect egg production and fertility parameters whereas it reduced ROS content and steroidogenesis in yolk egg. Furthermore, it ameliorated egg quality by decreasing the number of double-yolk eggs and by improving the size of normal eggs and the elasticity of the shell. Taken together, our data suggest the possibility of using dietary maternal GSE to improve egg quality.

## Introduction

Since several decades, breeders hen became the most efficient meat producing animals due to intense genetic selection for fast growth and high muscle yields. However, these changes led to a lot of problems in the management of broiler breeder hens such an increased body fat deposition and high incidence of metabolic and skeletal disorders. Moreover, reproductive dysfunctions occurred due to a negative correlation between muscle growth and reproduction effectiveness [1–3]. Nowadays, a restricting feed intake to approximately 60% of *ad libitum* is usual practice to prevents obesity, reduce mortality and to improve egg production in broiler hens [4]. However, this nutritional practice leads to problems of animal welfare including physiological stress, stereotypies and aggression in poultry [5]. Scientific literatures reported that there is an increased interest in the use of eco-friendly materials such as phyto-products from medicinal plants, fruits and herbal-based extracts [6]. Indeed, these compounds contain many bioactive phytochemicals which have been found to exhibit many properties such anti-inflammatory and antioxidant properties that may have beneficial effects on appetite, growth, immune status and reproductive functions [7]. In mammals, several *in vitro* and *in vivo* studies performed in male used a supplementation of natural antioxidants from plant origin as a means of counteracting the oxidative stress to improve fertility [8]. Even though fewer studies are available in the female, secondary metabolites from plants such as polyphenols have been reported to improve the number and quality of oocytes mice and humans [9, 10].

In broiler hens, we recently showed that grape seed extracts (GSE) maternal dietary supplementation reduces plasma and tissue oxidative stress associated to modulation of adipokines content in plasma and peripheral tissues [11]. Grape seeds are proanthocyanidins, mainly composed of monomeric catechin and epicatechin, gallic acid, and polymeric and oligomeric proanthocyanidins [12] which have been demonstrated to be more powerful free radical scavengers than vitamins C, E, and β-carotene [13]. The effects of grape seed extracts (GSE) have been largely investigated in cancer and metabolic cells [14] [15] but very few studies have been carried in ovarian cells. In mammals, GSE exerts *in vivo* beneficial effects on oxidative stress and on many metabolic disorders including insulin resistance [16] that could be associated to modulations of plasma adipokines such as adiponectin [17]. Recently, we showed that GSE treatments reduced oxidative stress (OS) and improved *in vitro* steroidogenesis without affecting cell proliferation and viability in human granulosa cells [18]. In hens, grape seed proanthocyanidin extract could prevent the ovarian aging process by reducing oxidative stress [19] and could provide some protection against the reproductive toxicity induced by the Cadmium endocrine disruptor [20]. However, no studies investigated the *in vivo* effects of the GSE on the egg performance (quantity and quality) and on the fertility parameters. The ovary of the reproductively active hen represents an interesting model for studying follicular development. It consists of small prehierarchical and maturing preovulatory follicles showing a hierarchy according to their size (F5/6 to F1; [21]. Moreover, as in women, ovarian functions in chicken are also regulated by gonadotropins, including FSH (follicle-stimulating hormone), LH (luteinizing hormone), and ovarian steroids.

Thus, our present study aimed to determine the *in vivo* effects of maternal GSE dietary supplementation on egg production and quality, reactive oxygen species (ROS) levels and steroid composition in yolk and fertility parameters. We also investigated a potential association between plasma adipokines and yolk ROS levels. All these analyses were performed in both experiments. In the first experiment, GSE dietary supplementation was carried out from hatching while in the second one it was performed in 4 weeks-old breeder hens.

## Materials and methods

### Ethical issues

An ethics committee “Comité d’Ethique en Expérimentation Animale Val de Loire » (CEEA VdL N°19) protocol registered approved all experimental studies, which were in accordance with the French National Guidelines for the care and use of animals for research purposes (certificate of authorisation to experiment on living animals APAFIS number 10237-201706151202940v3).

### Animals

Three hundred and twenty-four broiler breeder females chicks from Hendrix Genetics (Saint Laurent de la Plaine, France) were studied from day 1 to 40 weeks of age. Animals were divided in homogeneous groups of 10 to11 birds in 32 pens, each pen with an area of 3m^2^. The animals were reared at “Pôle Expérimental Avicole de Tours” (INRA, Nouzilly, France) according to the conventional conditions of breeding: 24h of light on arrival, day length being reduced to approximately 8h at two days of age, then kept constant until the age of photostimulation (21^st^ week). From 21 weeks of age, there was a gradual increase in exposure to light up to 15h per day at 25 weeks. Animals were maintained under this light regime until the end of the experiment and then they were killed by electrical tunning and bled out as recommended by the ethical committee.

### Diets composition

From one to the fourth week of age, female breeder chicks received an *ad libitum* diet (free access to food), called a starting diet. At week 4, all animals received a restricted diet according to Hendrix Genetics recommendation. From 4 week to 40 week, animals received three different diets: growing (from 4 to 18 week), before laying (from 18 week to 21 week) and laying (from 21 week to 40 week). We performed two experiments according the time of GSE supplementation as described below. For both experiment, remaining feed was weighed. The composition of GSE used for this study was analysed by HPLC (High Performance Liquid Chromatography). The most important component was the procyanidins (> 90%). The supplement was manually mixed with the diet at the proper concentration of the total diet. The composition of the diets is shown in the S1 Table and a schema of the experimental design is showed in S1 Fig.

### Experiment 1

In the experiment 1, the animals were divided into three groups: group A (control, n = 92), group B and C supplemented with GSE at 0.5% (n = 80) and 1% (= 80) of the total diet composition, respectively, since the age of 4 weeks until 40 week-old.

### Experiment 2

In the experiment 2, we used two groups of animals: group A (control, n = 92) and group D (supplemented with 1% of GSE since the hatching until 40 week-old, n = 72).

### Measurement of egg production and quality

In each experiment, from the 24^th^ week, the eggs from each pen were collected twice a day, counted and weighted using a balance (Ohauss, Pionner) The numbers of normal and double (eggs with two egg yolks) eggs were collected and counted. The weight of the albumen, the egg yolk and the dehydrated shell were determined. At the beginning of the laying period (26^th^ week), the width and the length of the eggs laid for each group of animals were measured using a digital calliper (Mitutoyo, CD-20DCX). Using an Instron instrument (Instron, UK527), we analysed the static stiffness (Sd, N/mm), the tensile strength (F), the elastic modulus (Eshell, N/mm^2^), the toughness (Kc, N/mm^3^) of the eggs. The weight and the thickness of the shell were also measured. These parameters were assessed for 30 eggs of each group of animals in each experiment.

### Determination of weight and number of follicles

In each experiment, at 40 weeks old, 10 hens per group were selected and the preovulatory follicles (from 1 to 4) were collected, hierarchically defined and weighed. Granulosa cells and theca cells were collected from the F1.

### Fertility parameters

For artificial insemination, we used the semen of 48 cocks (Cobb500), collected and pooled to form a single sample. For each experiment, the hens were inseminated with 2 x 10^8^ spermatozoa, at 28^th^ and 33^rd^ week. Eggs were collected and counted daily for 3 weeks following the artificial insemination and incubated every seven days. We assessed the number of unfertilised eggs, early (EEM) and late (LEM) embryonic mortality by breaking eggs and candling on the 7^th^ (EEM) and 14^th^ (LEM) day of incubation. The different percentages (EEM, LEM, hatchability, hatchability of fertile eggs and fertility) were calculated using the following formulae:

%EEM=numberofEEM/(numberofincubatedeggs–unfertilisedeggs)*100

%LEM=numberofLEM/(numberofincubatedeggs–unfertilisedeggs+numberofEEM)100

%Hatchabilityset=(numberofhatchedchicks/numberofincubatedeggs)*100

%Hatchabilityoffertileeggs=(numberofhatchedchicks/numberoffertileeggsafter14daysofincubation)*100

### Measurement of progesterone, androstenedione, testosterone and oestradiol deposition in egg yolk

In each experiment, we assessed steroids concentration from 30 egg yolks per group. Steroids were extracted with diethyl ether after intense agitation and centrifugation. The steroid-containing diethyl ether phase was decanted after freezing the tubes in nitrogen for 10s. The organic solvents were then evaporated and the extracts taken up in phosphate buffer. Steroid hormones (progesterone, oestradiol, testosterone and androstenedione) were then measured in the extracts using ELISA assays. For progesterone, the ELISA assay was performed as described by [22]. The sensitivity of the assay was 0.4 ng/mL. Oestradiol and testosterone concentrations were determined using commercial ELISA assays from Cayman Chemicals and the sensitivity of these assays was 0.01 ng/mL. Androstenedione levels were analysed using an ELISA assay from Abcam and the sensitivity of the assay was 0.01 ng/mL. The intra-assay and inter-assay coefficients of variation (CV) for each assay averaged <10%.

### Measurements of the expression of steroidogenic genes in granulosa cells

In each experiment, total RNA from granulosa cells of preovulatory follicle 1 of ten animals per group was extracted by homogeneisation in the TRIzol tissue reagent using an ULTRATURAX instrument, according to the manufacturer’s recommendations (Invitrogen, by Life Technologies, Villebon sur Yvette, France). The cDNA was generated by reverse transcription of total RNA (2 μg) in a mixture comprising 0.5 mM of each deoxyribonucleotide triphosphate (dATP, dTTP, dGTP, dCTP), 2 M of RT Buffer, 15 μg/μL of oligodT, 0.125 U of ribonuclease inhibitor, and 0.05 U of Moloney murine leukemia virus reverse transcriptase (MMLV) for one hour at 37°C. Real-time PCR was performed using the MyiQ Cycle Device (Bio-Rad, Marnes-la-Coquette, France), in a mixture with SYBR Green Supermix 1X Reagent (Bio-Rad, Marnes-la-Coquette, France), 250 nM specific primers (Invitrogen by Life Technologies, Villebon-sur-Yvette, France) and 3 μL of cDNA diluted 1:5 in water) for a total volume of 11 μL. The samples were set up in duplicate on the same plate according to the following procedure: after an incubation of 2 min at 50°C and a denaturation step of 10 min at 95°C, samples were subjected to 40 PCR cycles (30 s at 95°C, 30 s at 60°C, 30 s at 72°C). The primers used are shown in the S2 Table. For each gene, the relative abundance of transcription was determined by calculating e^-ct^. Then, the relative expression of the gene of interest was related to the relative expression of the geometric mean of the three reference genes (GAPDH, ACTB and EIF3).

### In vitro culture of hen granulosa cells

In each experiment, granulosa cells from preovulatory follicles 1 (F1) of hens fed with GSE dietary supplementation or with a control diet were collected from 7 animals for each group. Then, cells were dispersed in 0.3% collagenase type A (Roche Diagnostic, Meylan, France) in F12 medium containing 5% foetal bovine serum (FBS), at 37°C. Cells were pelleted by centrifugation, washed twice with fresh medium and counted in a haemocytometer. The viability of granulosa cells was estimated by Trypan Blue exclusion. Cells were cultured in a medium composed of DMEM supplemented with 100 U/mL penicillin, 100 mg/L streptomycin, 3 mmol/L L-glutamine and 5% FBS. The cells were initially cultured for 24h with no treatment. After overnight serum deprivation, cells collected from hens Rhode Island were stimulated with GSE (0.01, 0.1, 1, 50 and 100 μg/mL) or left untreated for 48h. Cells collected from hens fed with GSE supplementation were stimulated with IGF1 (10^-8^M), LH (10^-8^M), IGF1+LH (10^-8^M) or left untreated. All cultures were maintained under a water-saturated atmosphere of 95% air/ 5% CO2 at 37°C.

### In vitro measurement of progesterone secretion by granulosa cells

The concentration of progesterone secreted into the medium by granulosa cells under various conditions was determined according to an ELISA protocol described by [22]. The sensitivity of the kit was 0.4 ng/mL. The intra- and inter-assay coefficients of variation were <10% and <4.3% respectively. This experiment was carried out using four replicates of three hens for each group.

### Adipokine assays

Plasma concentration of three adipokines were determined using ELISA assays. Chicken-specific kits, MBS269004 (sensitivity 5 pg/mL), MBS016609 (sensitivity 0.1 μg/mL) and MBS738819 (sensitivity 0.1 ng/mL), were used for NAMPT, ADIPOQ and RARRES2 respectively (My BioSource). The experiment was performed following the manufacturer’s protocol with an intra-assay coefficient of variation ≤ 8%, < 15% and < 5.6 respectively. The absorbance was measured at 450 nm and then compared with reference values.

### Measurement of ROS (hydrogen peroxide (H₂O₂) in yolk

The contents of ROS, hydrogen peroxide (H₂O₂) from 10 egg yolks per group in each experiment were measured by Ros-Glo H2O2 assay (Promega Corporation, Charbonnières-les-Bains, France). In each analysis, one gram of each egg yolk for each group was precisely analysed following the manufacturer’s protocol.

### Statistical analysis

Data are represented as mean ± s.e.m., with a level of significance less than 0.05 (*P < 0.05). For both experiment, an analysis of variance using repeated measurements (Proc.Mix procedure) was used to compare the average numbers of normal eggs among the different hen groups. An analysis of variance (Proc.GLM procedure) was used to compare the number of double-yolk eggs, the weight of follicles, the content of ROS, the average concentrations of secreted steroids and androgen and levels of expression of adipokines and their receptors among the different groups. For both experiments, we analysed s the GSE supplementation effect (diet effect). A chi-square test was used for analysis of percentage fertility between the different parameters. A Pearson test was used to analyse correlations between ROS content and steroid in yolk egg and plasma adipokine concentration. The correlation was noted ‘*r*’ and the *P* value was considered significant if *P* < 0.05. SAS software (version 9.3, Cary, USA) was used for all analyses. Different letters indicate significant differences (*P* < 0.05).

## Results

### Effect of GSE maternal dietary supplementation on the egg production and quality (Fig 1A, Table 1)

**Fig 1:**
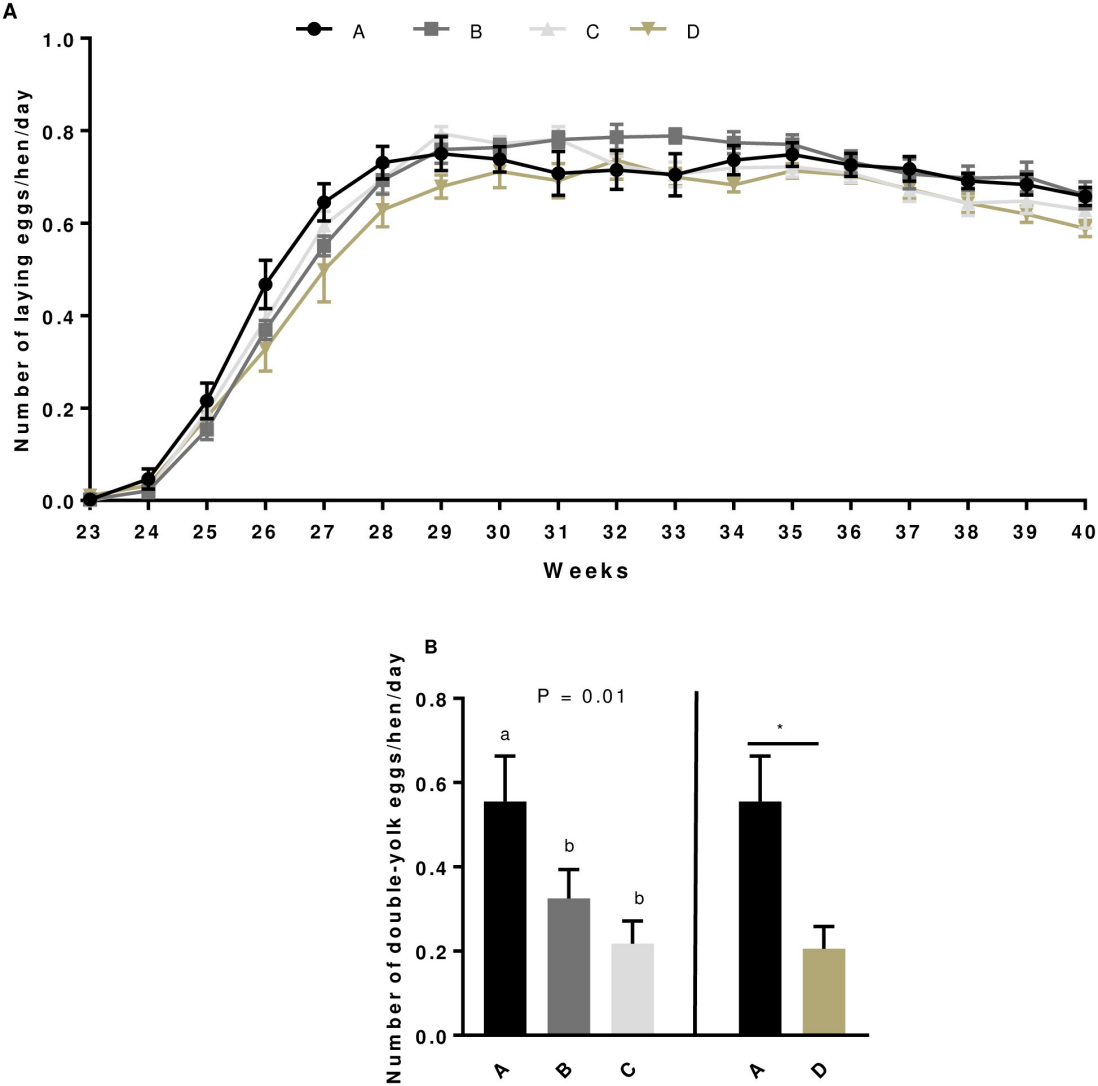
Laying curve (A) and number of double-yolk eggs (B) in broiler hens from 23^rd^ to 40^th^ week. Eggs were collected and weighed daily for all hens fed with different concentrations of GSE dietary supplementation or with a control diet. A: animals fed with control diet without GSE supplementation (n = 92), B and C: animals supplemented with GSE at 0.5% and 1% of the total diet composition, respectively, starting at 4 week-old until 40 week-old (n = 80), and D: supplementation with GSE at 1% of the total diet composition starting at hatch until 40 week-old (n = 72). Results are presented as lsmeans ± S.E.M. *P* values and different individual letters indicate a significant effect of the diet between A, B and C groups or between A and D groups. *P* value was considered as significant if *P* < 0.05.

**Table 1:** Number and quality of laying eggs of broiler hens fed with a GSE diet supplemented. Group A: animals fed with control diet without GSE supplementation, B and C: animals supplemented with GSE at 0.5% and 1% of the total diet composition, respectively, starting at 4 week-old until 40 week-old and D: supplementation at 1% of the total diet composition starting at hatch until 40 week-old.

Parameters	A	B	C	D	Diet ABC Exp.1	Diet AD Exp.2
Number of normal laying egg /hen/day	0.59 ± 0.21	0.59 ± 0.24	0.58 ± 0.23	0.55 ± 0.26	**0.85**	**0.16**
Number of double-yolk eggs /hen/day	0.56 ± 0.11 ^a^	0.33 ± 0.07 ^b^	0.22 ± 0.05 ^b^	0.21 ± 0.05	**0.01**	**0.02**
Age for the first egg (week)	24.25 ± 0.25	24.57 ± 0.3	24.29 ± 0.18	24.2 ± 0.37	0.62	0.91
Age at the laying peak (week)	29.38 ± 0.63	31.71 ± 0.8	29.86 ± 0.4	30.8 ± 0.8	**0.04**	0.19
Egg weight (g)	52.37 ± 0.57 ^a^	53.88 ± 0.6 ^a^	54.59 ± 0.62 ^b^	53.61 ± 0.79	**0.02**	0.2
Yolk weight (g)	14.02 ± 0.15	14.35 ± 0.23	14.39 ± 0.21	14.13 ± 0.24	0.17	0.69
Albumen weight (g)	30.27 ± 0.43	31.12 ± 0.84	31 ± 0.53	30.51 ± 0.63	0.41	0.75
Length (mm)	50.79 ± 0.5 ^a^	54.68 ± 0.32 ^b^	55.33 ± 0.26 ^b^	54.84 ± 0.27	**< 0.0001**	**< 0.0001**
Width (mm)	38.09 ± 0.401 ^a^	42.04 ± 0.2 ^b^	41.74 ± 0.23 ^b^	41.72 ± 0.3	**< 0.0001**	**< 0.0001**
% of shell	9.36 ± 0.13	9.24 ± 0.16	9.34 ± 0.11	9.09 ± 0.14	0.83	0.18
Thickness (mm)	0.319 ± 0.01	0.322 ± 0.01	0.322 ± 0.004	0.312 ± 0.01	0.87	0.32
Toughness (N/mm3)	486.01 ± 7.7	481.17 ± 8.45	470.37 ± 10.78	463.17 ± 11.14	0.44	0.09
Eshell (N/mm^2^)	16640 ± 357.55 ^a^	18546 ± 391.05 ^b^	18659 ± 450.15 ^b^	17738 ± 507.53	**0.0003**	0.07
Tensile strengh (N)	32.93 ± 0.84	34.52 ± 0.91	33.53 ± 0.78	31.28 ± 1.02	0.47	0.22
Static stiffness (N/mm)	156.9 ± 4.05	164.5 ± 3.91	157.2 ± 4.3	152.2 ± 5.98	0.42	0.5

The number of laying egg was determined in the whole period from 23 to 40^th^ week including all animals and the quality of eggs was determined at 26^th^ week on 30 eggs from each group of animals. Results are presented as lsmeans ± SEM. *P* values and different individual letters indicate a significant effect of the diet between A, B and C groups or between A and D groups. *P* value was considered as significant if *P* < 0.05.

In the experiment 1 (group A,B and C) and experiment 2 (group A vs D), the number of normal laying eggs per hen per day, the age at the first egg or at the laying peak were unaffected by the GSE dietary supplementation (Table 1 and Fig 1A). As shown in Table 1, the number of normal laying eggs per hen and per day in whole period (from week 23 to week 40) was similar between the different groups of animals whereas that of double laying eggs was significantly reduced by the GSE maternal dietary supplementation (Fig 1B). Indeed, GSE dietary supplementation reduced the number of double egg in B, C and D groups as compared to the control (B: 0.33 ± 0.07; C: 0.22 ± 0.05; D: 0.21 ± 0.05 vs A: 0.56 ± 0.11; *P* < 0.05). At week 26, we assessed different parameters concerning the quality of eggs (Table 1). In experiment 1, we noted that the weight of eggs was significantly increased in C group of animals (*P* < 0.02) and this was associated with a higher length (*P* < 0.0001) and width (*P* < 0.0001) of eggs without any variation in the yolk and albumen weight (Table 1). On the other hand, the Eshell corresponding to the elasticity of the shell was significantly higher in the B and C groups versus control animals (*P* = 0.0003) whereas it was unaffected in the experiment 2 (group D compared with the control animals (Table 1)). The percentage of shell, the toughness and thickness of the shell, the tensile strength and the static stiffness were similar in all GSE supplemented group and control group whatever the experiment (Table 1).

### Effect of GSE supplementation on weight of follicles and fertility parameters

**Fig 2:**
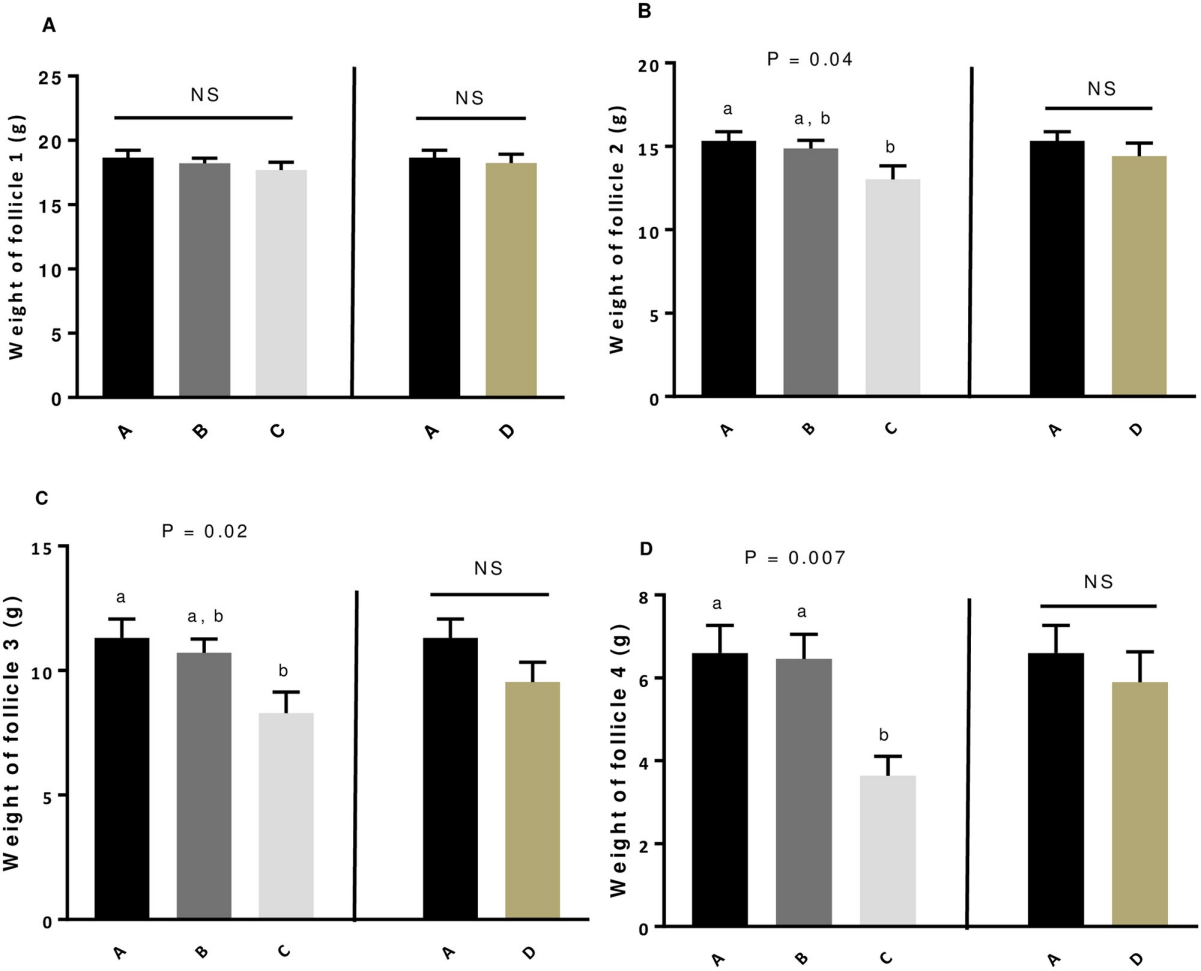
Weight of preovulatory follicles F1 (A), F2 (B), F3 (C) and F4 (D) of broiler hens fed with different concentrations of GSE supplementation. Follicular hierarchy of 10 animals selected randomly from each group (A to D) was analysed at the 40th week. Each preovulatory follicle from each animal was weighed. A: animals fed with control diet without GSE supplementation, B and C: animals supplemented with GSE at 0.5% and 1% of the total diet composition, respectively, starting at 4 week-old until 40 week-old, and D: supplementation with GSE at 1% of the total diet composition starting at hatch until 40 week-old. Results are presented as lsmeans ± S.E.M. *P* values and different individual letters indicate a significant effect of the diet between A, B and C groups (experiment 1) or between A and D (experiment 2) groups. *P* value was considered as significant if *P* < 0.05.

We weighed the preovulatory follicles from 1 to 4 (F1 to F4), for 10 animals per group of animals. In both experiment, the GSE dietary supplementation did not affect the weight of F1 (Fig 2A). Only in the experiment 1, the supplementation of 1% GSE from week 4 significantly reduced the weight of the preovulatory follicle 2 (*P* = 0.04, Fig 2B), follicle 3 (*P* = 0.02, Fig 2C) and 4 (*P* = 0.007, Fig 2D) as compared to other group of animals. After the first artificial insemination (AI) at 28 weeks, we assessed the fertility parameters (Table 2). The GSE supplementation did not affect the EEM, LEM, the hatchability of incubated or fertile eggs and the fertility rate whatever the experiment (Table 2). For all fertility parameters, we found similar data for the second AI (33 weeks).

**Table 2:** Percentage of unfertilised eggs, early (EEM) and late (LEM) embryonic mortality and fertility after artificial insemination in broiler hens fed with GSE supplementation.

Parameters	A	B	C	D	Diet ABC	Diet AD
% Unfertilised	5.89 ± 0.89 ^a^	4.23 ± 0.65 ^b^	4.06 ± 0.75 ^b^	6.28 ± 0.99	0.40	0.98
% EEM	4.62 ± 0.69	4.49 ± 0.55	5.46 ± 0.65	3.89 ± 0.54	0.16	0.73
% LEM	1.29 ± 0.47	1.2 ± 0.28	1.33 ± 0.33	1.12 ± 0.37	0.98	0.81
% Fertility	88.8 ± 1.19	90.53 ± 0.76	89.73 ± 1.11	89.17 ± 1.15	0.93	0.92
% Hatchability of incubated eggs	79.88 ± 1.04	79.55 ± 0.99	79.87 ± 1.15	77.5 ± 1.38	0.99	0.67
% Hatchability of fertile eggs	90.05 ± 0.64	87.93 ± 1.01	89.05 ± 0.83	86.86 ± 0.88	0.94	0.6

Results are presented as lsmeans ± SEM. *P* values and different individual letters indicate a significant effect of the diet between A, B and C groups (experiment 1) or between A and D groups (experiment 2). P value was considered as significant if *P* < 0.05. EEM, early embryo mortality; LEM, late embryo mortality. A: animals fed with control diet without GSE supplementation, B and C: animals supplemented with GSE at 0.5% and 1% of the total diet composition, respectively, starting at 4 week-old until 40 week-old and D: supplementation at 1% of the total diet composition starting at hatch until 40 week-old.

### Effect of GSE supplementation on steroids hormones concentration in egg yolks and mRNA expression of steroidogenic enzymes in granulosa cells

**Fig 3:**
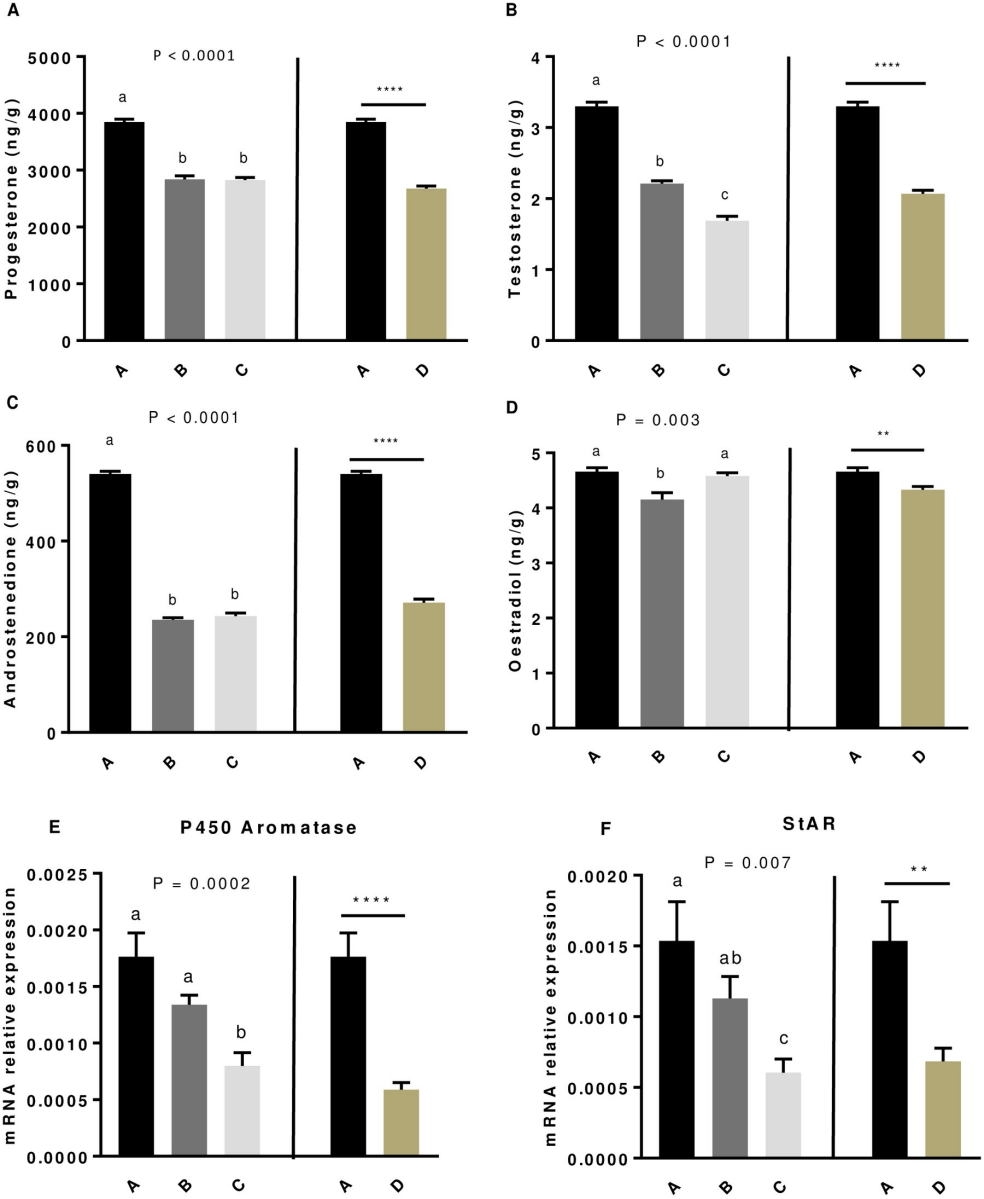
Levels of progesterone (A), testosterone (B), androstenedione (C) and oestradiol (D) in egg yolk and P450 aromatase (E) and StAR (F) mRNA expression in granulosa cell from broiler hens fed with different concentrations of GSE. Steroids were extracted from 30 egg yolks for each group, at 33 weeks-old. mRNA was extracted from granulosa cells of 10 preovulatory follicles for each group. A: animals fed with control diet without GSE supplementation, B and C: animals supplemented with GSE at 0.5% and 1% of the total diet composition, respectively, starting at 4 week-old until 40 week-old (experiment 1) and D: supplementation with GSE at 1% of the total diet composition starting at hatching until 40 week-old (experiment 2). Results are presented as lsmeans ± S.E.M. *P* values and different individual letters indicate a significant effect of the diet between A, B and C groups (experiment 1) or between A and D groups (experiment 2). *P* value was considered as significant if P < 0.05. **P < 0.005 and **** *P* < 0.0001.

As shown in Fig 3 and Table 3, the production of progesterone (Fig 3A), testosterone (Fig 3B) and androstenedione (Fig 3C) in the egg yolks was significantly lower (*P* < 0.0001) for all dietary GSE supplemented groups compared to the control group whatever the experiment. In addition, we noticed that oestradiol concentration in the egg yolks was reduced only for B (*P* = 0.0003) and D (*P* = 0.001) groups compared to the control (Fig 3D). We next examined whether this decrease in steroid concentration in egg yolks in response to a GSE dietary supplementation was a result of some key enzymes of steroidogenesis (3β-HSD, P450scc, P450 aromatase) and/or of StAR, an important cholesterol carrier in the granulosa cells from F1 preovulatory follicle. As shown in Fig 3E and 3F, we showed that the mRNA expression of P450 aromatase (Fig 3E) and StAR (Fig 3F) was significantly reduced in C and D groups compared to the control group (*P* < 0.0001) whereas the mRNA expression of the other enzymes (3β-HSD and P450scc) was unaffected.

**Table 3:** Progesterone, testosterone, androstenedione and oestradiol concentrations in egg yolk at 33 weeks-old.

Period	Group	Progesterone (ng/g)	Testosterone (ng/g)	Androstenedione (ng/g)	Oestradiol (ng/g)
**33 weeks**	A	3846 ± 53.47 ^a^	3.3 ± 0.06 ^a^	540.5 ± 5.26 ^a^	4.66 ± 0.07 ^a^
	B	2839 ± 63.82 ^b^	2.21 ± 0.04 ^b^	235.2 ± 4.45 ^b^	4.15 ± 0.13 ^b^
	C	2827 ± 45.1 ^b^	1.69 ± 0.06 ^c^	243.4 ± 6.21 ^b^	4.58 ± 0.06 ^a^
	D	2679 ± 42.47	2.07 ± 0.05	271.2 ± 7.54	4.33 ± 0.06
*P*	Diet ABC	**<0.0001**	**<0.0001**	**<0.0001**	**0.0003**
*P*	Diet AD	**<0.0001**	**<0.0001**	**<0.0001**	**0.001**

Results are presented as lsmeans ± SEM. *P* values and different individual letters indicate a significant effect of the diet between A, B and C groups (experiment 1) or between A and D groups (experiment 2). *P* value was considered as significant if P < 0.05. Thirty eggs yolk were collected from broiler hens fed with different concentrations of GSE dietary supplementation or with a control diet. A: animals fed with control diet without GSE supplementation, B and C: animals supplemented with GSE at 0.5% and 1% of the total diet composition, respectively, starting at 4 week-old until 40 week-old (experiment 1) and D: supplementation at 1% of the total diet composition starting at hatch until 40 week-old (experiment 2).

### Effects of GSE dietary supplementation on *in vitro* steroidogenesis in hen primary granulosa cells

**Fig 4:**
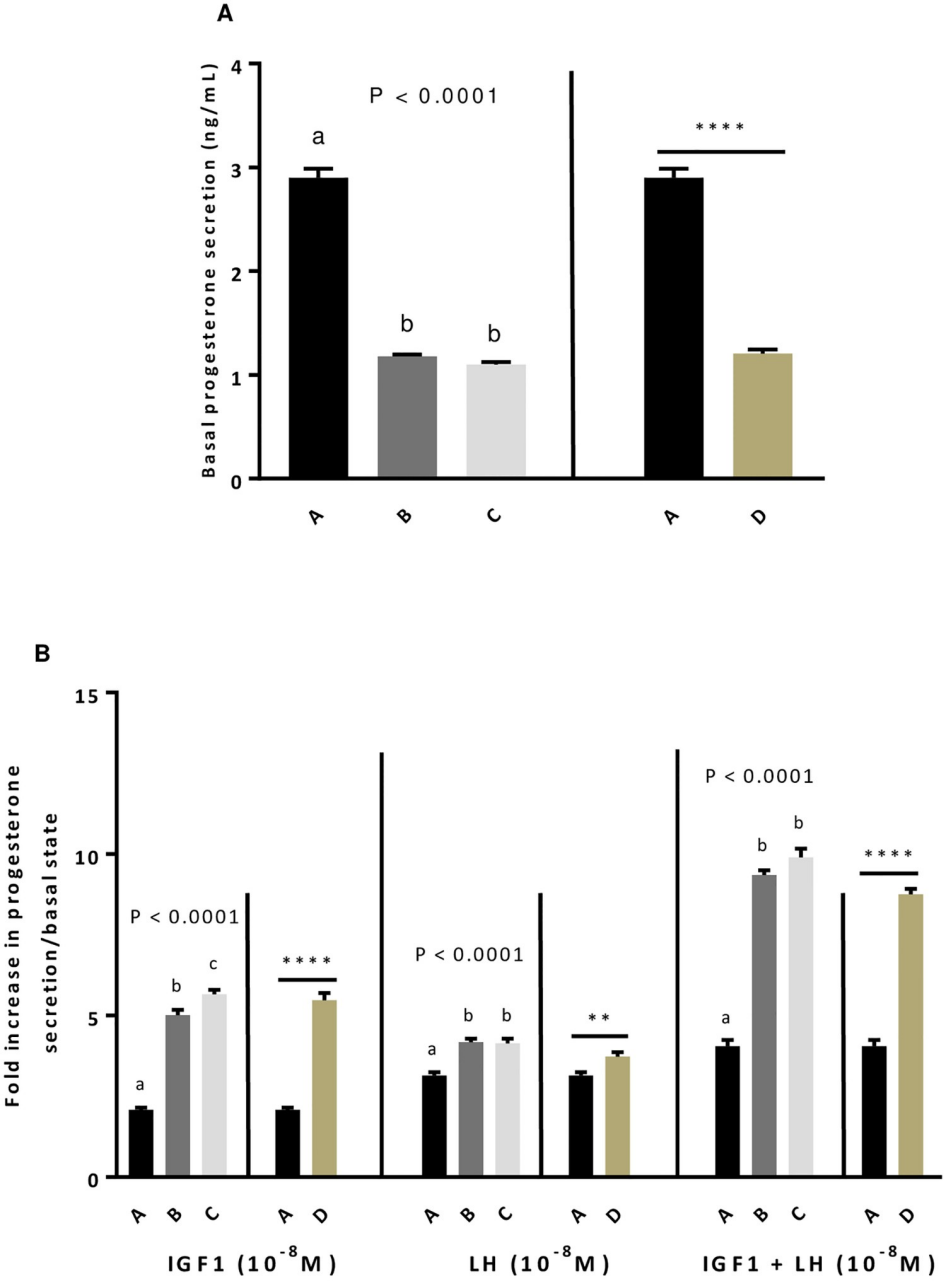
Effect of dietary GSE supplementation on *in vitro* steroidogenesis at basal state (A) and in response to IGF1 and LH alone or in combination in hen primary granulosa cells. Granulosa cells from preovulatory follicles 1 (F1) from different group of animals (A to D) were seeded for 24 h and after overnight serum starvation, granulosa cells were incubated with IGF1 (10^-8^M), LH (10^-8^M) or both for 48 h. The culture medium was then collected. Basal progesterone level (A) and the response to IGF1 or/and LH (B) were assessed. A: animals fed with control diet without GSE supplementation, B and C: animals supplemented with GSE at 0.5% and 1% of the total diet composition, respectively, starting at 4 week-old until 40 week-old, and D: supplementation with GSE at 1% of the total diet composition starting at hatch until 40 week-old. Results are presented as lsmeans ± S.E.M. *P* values and different individual letters indicate a significant effect of the diet between A, B and C groups (experiment 1) or between A and D groups (experiment 2). *P* value was considered as significant if *P* < 0.05. **P < 0.005 and **** *P* < 0.0001.

As observed for egg yolks, the GSE dietary supplementation whatever the experiment significantly decreased the production of progesterone by unstimulated primary granulosa cells from F1 preovulatory follicles (*P* < 0.0001, Fig 4A). However, after stimulation of cells with IGF1 (10^−8^ M) or LH (10^−8^ M) alone or in combination, the GSE dietary supplementation significantly increased the granulosa cells response suggesting a better sensitivity of cells to these hormones (*P* < 0.005, Fig 4B).

### Effect of GSE dietary supplementation on Reactive Oxygen Species (ROS) levels in egg yolks

**Fig 5:**
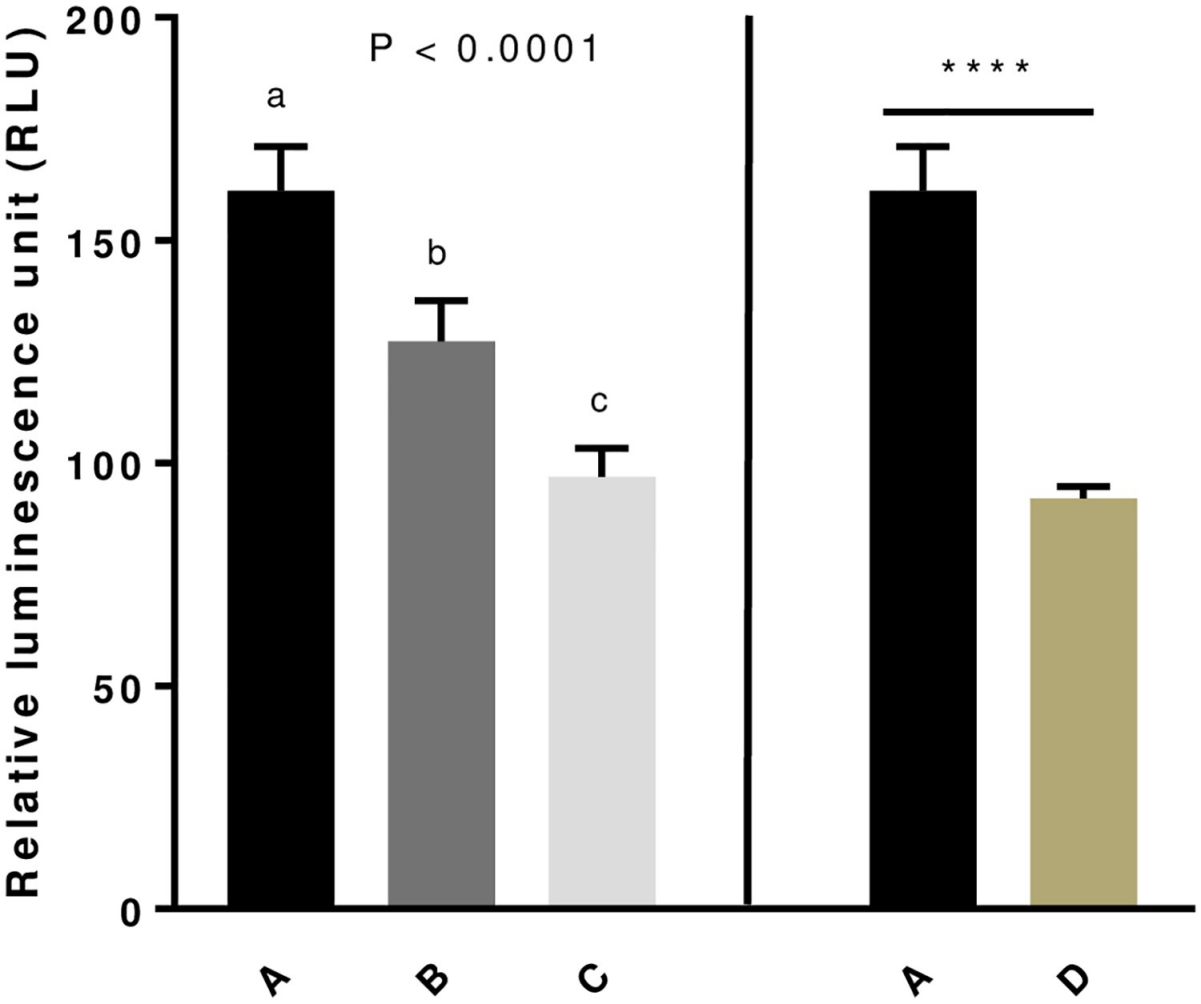
Level of Reactive Oxygen Species (ROS, H202) in egg yolk of broiler hens fed with different concentrations of GSE dietary supplementation. ROS level (H202) was assessed from 1 g of each egg yolk using Ros-Glo assay. Ten egg yolks were analysed for each group of animals. A: animals fed with control diet without GSE supplementation, B and C: animals supplemented with GSE at 0.5% and 1% of the total diet composition, respectively, starting at 4 week-old until 40 week-old (experiment 1), and D: supplementation at 1% of the total diet composition starting at the hatching until 40 week-old (experiment 2). Results are presented as lsmeans ± S.E.M. *P* values and different individual letters indicate a significant effect of the diet between A, B and C groups (experiment 1) or between A and D groups (experiment 2). *P* value was considered as significant if *P* < 0.05. **** *P* < 0.0001.

Since some studies demonstrated that GSE were powerful free radical scavengers [13], we consequently evaluated by using Ros-Glo H202 assay the ROS (H202) amount in egg yolks. As shown in Fig 5, the GSE supplementation significantly decreased the ROS levels (*P* < 0.0001) in eggs yolks whatever the beginning of the maternal dietary supplementation period (hatching (D group, experiment 2) or week 4 (B and C group, experiment 1)). In addition, in experiment 1, when the GSE supplementation was applied at week 4, the ROS levels were lower in C as compared to B group showing a dose dependent decrease (Fig 5).

### Effect of the GSE dietary supplementation on adipokines plasma levels and association with steroids hormones and ROS amount in egg yolks

**Fig 6:**
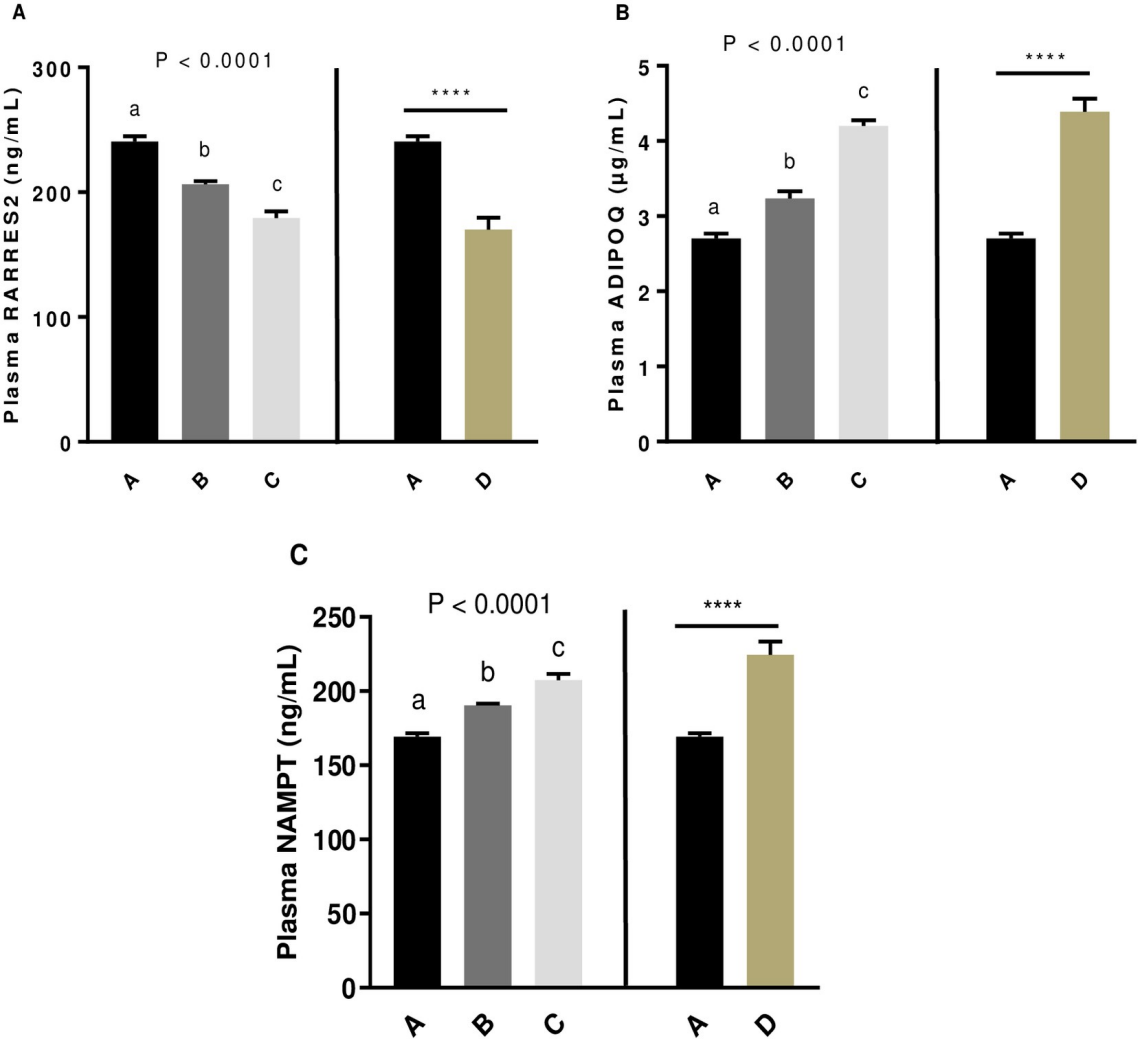
Level of plasma RARRES2 (A), ADIPOQ (B) and NAMPT(C) concentrations of broiler hens fed with different concentrations of GSE dietary supplementation. At 33^rd^ week, plasma adipokines concentration was assessed using specific ELISA assay in 11 animals from each group of animals. A: animals fed with control diet without GSE supplementation, B and C: animals supplemented with GSE at 0.5% and 1% of the total diet composition, respectively, starting at 4 week-old until 40 week-old, and D: supplementation at 1% of the total diet composition starting at hatch until 40 week-old. Results are presented as lsmeans ± S.E.M. *P* values and different individual letters indicate a significant effect of the diet between A, B and C groups (experiment 1) or between A and D groups (experiment 2). *P* value was considered as significant if *P* < 0.05. **** *P* < 0.0001.

Since we previously showed that plasma adipokines could be associated to fertility parameters in hens [23], we assessed the plasma concentration of RARRES2 (Fig 6A), ADIPOQ (Fig 6B) and NAMPT (Fig 6C) at 33 weeks before the second AI. The plasma RARRES2 concentration significantly decreased whereas plasma ADIPOQ and NAMPT concentrations increased in all GSE dietary supplemented groups compared to the control (*P* < 0.0001) in both experiment 1 and 2. Then, we assessed the correlation between these plasma adipokines and the steroids hormones in yolk eggs (Table 4). The correlation, was performed with A, B and C groups (experiment 1) and A and D (experiment 2). In both experiments, progesterone, androstenedione and testosterone concentrations in egg yolks were positively correlated with plasma RARRES2 (A,B, and C: r = 0.73, r = 0.79 and r = 0.85, respectively, *P* < 0.0001; A and D: r = 0.78, r = 0.86, r = 0.78, respectively, *P* < 0.0001) whereas they were negatively correlated with plasma ADIPOQ (A, B and C: r = -0.71, r = -0.7 and r = -0.86, respectively, *P* < 0.0001; A and D: r = -0.86, r = -0.9 and r = -0.88, respectively, *P* < 0.0001) and plasma NAMPT (A, B and C: r = -0.77, r = -0.76 and r = -0.83, respectively, *P* < 0.0001; A and D: r = -0.76, r = -0.82 and r = -0.77, respectively, *P* < 0.0001). Any correlation was significant for plasma oestradiol concentration. Moreover, we observed that ROS levels in egg yolks were positively correlated with plasma RARRES2 (A B and C: r = 0.81, *P* < 0.0001; A and D: r = 0.85, *P* < 0.0001) and negatively correlated with plasma ADIPOQ (A, B and C: r = -0.69, *P* < 0.0001; A and D: r = -0.77, *P* < 0.0001) and plasma NAMPT (A, B and C: r = -0.72, *P* < 0.0001; A and D: r = -0.71, *P* < 0.0001).

**Table 4:** Pearson correlation coefficient (*r*) calculated between plasma adipokines (RARRES2, ADIPOQ, NAMPT) concentration and steroids hormones (progesterone, androstenedione, testosterone, oestradiol) and ROS concentration in egg yolk, for the groups A, B and C (experiment 1) and then, A and D (experiment 2).

**ABC Group**		**RARRES2**	**ADIPOQ**	**NAMPT**
**Progesterone**	*r*	0.73	-0.71	-0.77
	*P*-value	**<0.0001**	**<0.0001**	**<0.0001**
**Androstenedione**	*r*	0.79	-0.7	-0.76
	*P-*value	**<0.0001**	**<0.0001**	**<0.0001**
**Testosterone**	*r*	0.85	-0.86	-0.83
	*P*-value	**<0.0001**	**<0.0001**	**<0.0001**
**Oestradiol**	*r*	0.05	0.22	-0.06
	*P*-value	0.79	0.22	0.72
**Reactive Oxygen Species (ROS)**	*r*	0.81	-0.69	- 0.72
	*P*-value	**<0.0001**	**<0.0001**	**<0.0001**
**AD Group**		**RARRES2**	**ADIPOQ**	**NAMPT**
**Progesterone**	*r*	0.78	-0.86	-0.76
	*P*-value	**<0.0001**	**<0.0001**	**<0.0001**
**Androstenedione**	*r*	0.86	-0.9	-0.82
	*P-*value	**<0.0001**	**<0.0001**	**<0.0001**
**Testosterone**	*r*	0.78	-0.88	-0.77
	*P*-value	**<0.0001**	**<0.0001**	**<0.0001**
**Oestradiol**	*r*	0.4	-0.34	-0.3
	*P*-value	0.06	0.12	0.17
**Reactive Oxygen Species (ROS)**	*r*	0.85	-0.77	-0.71
	*P*-value	**<0.0001**	**<0.0001**	**<0.0001**

Values (r) and significance (*P*-value) of the correlations are indicated. Correlations were considered as significant if *P* < 0.05. Groups: A (no supplementation), B (supplemented with 0.5% of the total diet composition starting at 4 week-old until 40 week-old), C (supplemented with 1% of the total diet composition starting at 4 week-old until 40 weeks-old) and D (supplemented with 1% of the total diet composition starting at hatch until 40 week-old).

### Effect of the GSE dietary supplementation on the expression of adipokines and their receptors in granulosa and theca cells

In granulosa and theca cells from F1 preovulatory follicles, we determined the effect of the GSE dietary supplementation on the mRNA expression (Table 5) of all adipokines (RARRES2, ADIPOQ, NAMPT) and their receptors (CMKLR1, CCRL2, GPR1, ADIPOR1, ADIPOR2). There was no effect of GSE supplementation in RARRES2 expression in granulosa cells, however we observed a decrease in the mRNA expression of its receptor CCRL2 (*P* = 0.05) in the C group compared to the control in the experiment 1. In addition, we showed a decrease in GPR1 expression in all GSE dietary supplemented group as compared the control in both experiments (Table 5). In granulosa cells, ADIPOQ was undetectable. However, we detected a significant decrease of the expression of ADIPOR1 for the D group compared to the control (*P* < 0.05) in the experiment 2. Concerning the expression of NAMPT, a decrease was observed in all GSE supplemented group compared to the control group (*P* < 0.0005) in both experiments. In theca cells, we observed a decrease in GPR1 expression in D group as compared to the control (*P* = 0.05) in the experiment 2. There was not statistical difference for the other genes.

**Table 5:** mRNA expression of adipokines (RARRES2, ADIPOQ, NAMPT) and their receptors (CMKLR1, CCRL2, GPR1, ADIPOR1, ADIPOR2) in granulosa and theca cells from preovulatory follicle 1 (F1).

Tissues	Genes	A	B	C	D	Diet ABC	Diet AD
Granulosa cells F1	*RARRES2*	0.4 ± 0.09	0.32 ± 0.03	0.33 ± 0.04	0.3 ± 0.04	0.56	0.32
	*CMKLR1*	0.001 ± 2.10^-4a,b^	0.0014 ± 3.10^-4a^	5.10^−4^ ± 5.10^-5b^	6.10^−4^ ± 3.10^−4^	**0.04**	0.31
	*CCRL2*	0.003 ± 7.10^-4a^	0.003 ± 6.10^-4a^	6.10^−4^ ± 1.10^-4b^	0.001 ± 7.10^−4^	**0.02**	0.2
	*GPR1*	0.5 ± 0.12 ^a^	0.3 ± 0.03 ^a,b^	0.22 ± 0.02 ^b^	0.19 ± 0.02	**0.01**	**0.03**
	*ADIPOR1*	0.002 ± 4.10^−4^	0.002 ± 2.10^−4^	0.003 ± 5.10^−4^	0.001 ± 2.10^−4^	0.6	**0.01**
	*ADIPOR2*	0.008 ± 7.10^−4^	0.008 ± 6.10^−4^	0.01 ± 0.001	0.007 ± 0.001	0.05	0.66
	*NAMPT*	0.02 ± 0.002 ^a^	0.01 ± 0.001 ^b^	0.01 ± 0.001 ^b^	0.009 ± 0.001	**0.0003**	**0.0002**
Theca cells F1	*RARRES2*	0.41 ± 0.04	0.4 ± 0.04	0.49 ± 0.03	0.36 ± 0.03	0.19	0.33
	*CMKLR1*	0.11 ± 0.03	0.12 ± 0.02	0.16 ± 0.04	0.08 ± 0.02	0.51	0.38
	*CCRL2*	0.45 ± 0.1	0.49 ± 0.06	0.77 ± 0.11	0.45 ± 0.03	0.06	0.98
	*GPR1*	0.1 ± 0.02	0.14 ± 0.02	0.08 ± 0.01	0.05 ± 0.006	0.23	0.05
	*ADIPOQ*	0.18 ± 0.05	0.21 ± 0.04	0.33 ± 0.07	0.19 ± 0.05	0.14	0.88
	*ADIPOR1*	5.10^−4^ ± 1.10^−4^	5.10^−4^ ± 3.10^−4^	1.10^−4^ ± 4.10^−5^	4.10^−4^ ± 2.10^−4^	0.16	0.85
	*ADIPOR2*	0.002 ± 4.10^−4^	0.001 ± 3.10^−4^	0.001 ± 3.10^−4^	0.001 ± 4.10^−4^	0.45	0.48
	*NAMPT*	0.13 ± 0.03	0.16 ± 0.02	0.12 ± 0.02	0.09 ± 0.01	0.47	0.14

Results are presented as lsmeans ± SEM. *P* values and different individual letters indicate a significant effect of the diet between A, B and C groups (experiment 1) or between A and D groups (experiment 2). *P* value was considered as significant if *P* < 0.05.

## Discussion

This present study shows for the first time that an *in vivo* GSE maternal dietary supplementation very early (at hatching) or lately (at week 4) reduces significantly ROS levels and steroid secretion in yolk egg and this was associated to variations of plasma and ovarian cell adipokines in reproductive broiler hens. Furthermore, we showed that this GSE supplementation ameliorated egg quality by decreasing the number of double-yolk eggs and by improving the elasticity of the shell. These data were observed without any alterations in fertility parameters and egg production.

Our results indicate that a dietary GSE supplementation significantly reduces the ROS levels in egg yolk suggesting a reduction in both lipid peroxidation and OS. These data can be considered beneficial since yolk oxidation leads to malondialdehyde production (product of lipid peroxidation), that is a toxic substance with negative effects on human and animal health [24]. A significant reduction on lipid peroxidation and increased antioxidant capacity in egg yolk were found in laying hens fed with grape pomace flour, as observed by Kara et al. [25] [26] and Galli et al. [27] in laying hens fed grape pomace and curcumin, respectively. GSE are known to decrease oxidative stress and ROS in various tissues or cells under different conditions. For example, in rats, GSE treatment decreased oxidative stress damage in liver following bile duct ligation [28] or high fat diet [29]. In broiler chicken, grape seed proanthocyanidin extracts decreases oxidative damages induced by aflatoxins in splenic tissues [30]. Thus, maternal dietary GSE supplementation is able to scavenge free oxygen radical in the egg yolk as it has been demonstrated in mammalian and chicken tissue.

We did not observe any effect of the supplementation on the egg production rate or the egg weight during the whole laying period (23–40 weeks). However, the GSE supplementation reduced the incidence of double-yolk eggs. The double-yolked avian egg is a common physiological process in commercial species of poultry [31, 32]. Double-yolk eggs are formed when two F1 follicles ovulated within three hours of each other become enclosed in one egg [33], and estimated to occur in 4~12.5% of broiler breeder pullet eggs in the first 3 months of laying [34, 35]. Double-yolk eggs are considered as a loss to overall commercial hatcheries because of their relatively lower yolk fertility rate due to their smaller yolk size and markedly lower hatchability rate [31, 36]. Thus, maternal dietary GSE supplementation could have a beneficial for the breeders. Interestingly, we found that eggs from hens fed with the diet supplemented with 1% GSE from 4 week-old were heaver, bigger and larger compared to the control at 33 weeks and we noticed a higher elasticity of the shell for all supplemented group. Until now no studies investigated the GSE extract on the egg performance and quality. However, Sahin et al., 2010 reported no effects of dietary resveratrol supplementation on egg production or egg quality related to the shell, yolk and albumen in quails [37]. Resveratrol is an antioxidant located in whole grape and has properties similar to proanthocyanidin, the main component in grape seed [38].

Since the GSE supplementation reduced ROS levels in yolk and high levels of ROS are known to alter fertility, we expected a better fertility in supplemented animals. Furthermore, we could also expect a better hatchability since the increase in circulating oxygen rates during the hatching lead to a higher production of free radicals due to oxidative processes that may be deleterious for the chicks [39]. Several studies showed that a supplementation of antioxidant such as vitamin E but also GSE directly *in ovo* can improve hatchability. For example, Salary et al., 2014 found an improvement in broiler chick hatching from vitamin E supplemented eggs, compared with the non-supplemented group [40]. Hajati et al., 2014 studied *in ovo* supplementation of an antioxidant compound (grape seed extract together with vitamin C) and found better hatching results for chicks from supplemented eggs [41]. According to Surai et al., 2016, egg yolk antioxidants control oxidation through reducing or deactivating free radicals before they act on chick organ tissues [39]. A study conducted by Urso et al., 2015 also showed that vitamin E (VE) supplementation in the breeders’ feed enabled improvement of egg hatching rates and the overall state of quality among newborn chicks [42]. However, in the present study, we observed no significant effect on the embryo viability, hatchability or fertility rate. These data could be explained by several reasons; first the percentage of fertility of animals could be too high (almost 90%) to observe a beneficial effect, second the levels of ROS in yolk if important in fertility regulation in control group were not high enough to induce a deleterious effect on the fertilization process or embryo development, finally the time of GSE supplementation was too long. At the opposite in the literature, a dietary selenium (antioxidant) supplementation increased hatchability and reduced embryonic mortality rate [43, 44]. Furthermore, using *in ovo* injection (at embryonic day 6.5), GSE has been shown to protect chicken embryos against Cadmium, an endocrine disruptor [20].

We observed a reduction in steroid content in yolk egg and this was associated to a lower production of progesterone and a lower expression of StAR and P450 aromatase by granulosa cells. Some studies showed that the steroid hormones of maternal origin in avian egg yolks have a strong influence on offspring development [45, 46]. Thus, it will be interesting to follow the growth of chicks from hens fed with a diet supplemented with GSE. In vitro, we observed that F1 granulosa cells from hens fed with a diet supplemented with GSE were more sensitive to IGF1 and LH alone or in combination concerning progesterone production. Several studies showed that GSE treatment improves insulin sensitivity by increasing expression and activation of insulin receptor signalling components [47]. Since insulin receptors and IGF1 receptors share a high similarity of structure and intracellular signalling components [48], we can hypothesize that GSE treatment improves IGF1 receptor signaling in hen granulosa cells. No effect of GSE treatment on LH receptor has yet described in the literature. In basal state (no IGF1 and LH stimulation), progesterone secretion is lower in granulosa cells from hens fed with a diet supplemented with GSE. In a recent paper, we showed a positive effect of GSE treatment on progesterone and oestradiol secretion by cultured human granulosa cells [49]. This discrepancy of the results between the two studies could be explained by the fact that the hen granulosa cells were indirectly exposed to GSE treatment (through maternal diet). It will be interesting to determine the composition in polyphenols of the preovulatory follicles to determine which polyphenols are exposed to granulosa cells.

In plasma, we found that GSE treatment decreases RARRES2 and increases ADIPOQ and NAMPT. Furthermore, we observed that plasma RARRES2 was positively associated to ROS levels and steroid production in yolk egg. Opposite results were observed for plasma ADIPOQ and NAMPT. RARRES2 has been already described involved in the regulation of the OS. Indeed, RARRES2 increased ROS levels in C2C12 myoblasts [50] and upregulation of RARRES2 and one of these receptors, CMKLR1 may explain the imbalance of OS and apoptosis in the ovaries of obese mice [51]. In hen granulosa cells, maternal dietary GSE supplementation at week 4 significantly reduced CMKLR1 and GPR1 mRNA expression suggesting that RARRES2 signaling could participate to the reduction of ROS levels in yolk egg. The increase in plasma ADIPOQ in response to maternal diet GSE supplementation in hens is a good agreement with data obtained in mammals. Indeed, in rats, maternal intake of GSE induces an increase of ADIPOQ in plasma [52]. Moreover, ADIPOQ treatment significantly decreases the production of intercellular ROS [53]. NAMPT is involved in oxidative stress processes and is responsible for the production of NAD, which is involved in cellular redox reactions. The inhibition of NAMPT decreased cell growth and enhanced susceptibility to oxidative stress. NAMPT enhances ROS production in human vascular endothelial cells [54] and induces oxidative stress in differentiated C2C12 myotubes [55]. Furthermore, NAMPT expression can be modulated by GSE treatment in rat liver [56]. All these data suggest that plasma and ovarian expression of adipokines could participate to regulate the ROS levels and OS in yolk egg and ovarian cells in hens fed with a diet supplemented with GSE.

## Conclusions

In conclusion, we showed that a maternal diet GSE supplementation at hatching or at 4 week-old until 40 week-old significantly improved the quality of eggs by decreasing the number of double-yolk eggs and by increasing the shell elasticity without affecting the egg production and fertility parameters. Furthermore, GSE supplementation reduced the levels of ROS and steroidogenesis in yolk egg and this was associated to variations in plasma and ovarian cell expression of adipokines. Taken together, our data suggest the possibility of using dietary maternal GSE to improve egg quality. However, more experiments are necessary to investigate the effect of sequential maternal dietary GSE supplementation for a short time at specific period such as pre-laying on the fertility parameters and laying performance in adult offspring.

## Supporting information

S1 Fig Experimental protocol design. From one to the fourth week of age, 324 female breeder chicks received an *ad libitum* diet (free access to food), called a starting diet. At week 4, all animals received a restricted diet according to Hendrix Genetics recommendation. From 4 week to 40 week, animals received three different diets: growing (from 4 to 18 week), before laying (from 18 week to 21 week) and laying (from 21 week to 40 week). We performed two experiments according the time of GSE supplementation. In the experiment 1, the animals were divided into three groups: group A (control, n = 92), group B and C supplemented with GSE at 0.5% (n = 80) and 1% (= 80) of the total diet composition, respectively, since the age of 4 weeks until 40 week-old. In the experiment 2, we used two groups of animals: group A (control, n = 92) and group D (supplemented with 1% of GSE since the hatching until 40 week-old, n = 72). For both experiments, eggs were collected during the whole laying period, from 23^th^ to 40^th^ week. At 26^th^ week, the quality of 30 eggs per group was assessed. At 28^th^ week, the first Artificial Insemination (AI1) was performed and the fertility parameters were assessed after the hatching. At 33^th^ week, the steroidogenesis and ROS level in yolk eggs were assessed, for 30 and 10 egg yolks per group of animals, respectively, and then the second Artificial Insemination (AI2) was performed.
(PDF)

S1 Table Composition of the diet for the different groups of animals. A: control without supplementation, B and C: supplementation at 0.5% and 1% of the total diet composition, respectively, starting at 4 week-old until 40 week-old (experiment 1), and D: supplementation at 1% of the total diet composition, starting at hatch until 40 week-old (experiment 2).
(PDF)

S2 Table Oligonucleotide primer sequences. (PDF)

## References

[1] Chen SE, McMurtry JP, Walzem RL. Overfeeding-induced ovarian dysfunction in broiler breeder hens is associated with lipotoxicity. Poult Sci. 2006;85(1):70–81. 10.1093/ps/85.1.70 . 16493948

[2] Decuypere E, Bruggeman V, Everaert N, Li Y, Boonen R, De Tavernier J, et al The broiler breeder paradox: ethical, genetic and physiological perspectives, and suggestions for solutions. Br Poult Sci. 2010;51(5):569–79. 10.1080/00071668.2010.519121 . 21058058

[3] Richards MP, Proszkowiec-Weglarz M. Mechanisms regulating feed intake, energy expenditure, and body weight in poultry. Poult Sci. 2007;86(7):1478–90. 10.1093/ps/86.7.1478 . 17575199

[4] Leeson S, Summers JD, Caston LJ. Response of layers to dietary flaxseed according to body weight classification at maturity. Journal of Applied Poultry Research. 2000;9(3):297–302. 10.1093/japr/9.3.297

[5] Hocking PM, Maxwell MH, Mitchell MA. Relationships between the degree of food restriction and welfare indices in broiler breeder females. Brit Poultry Sci. 1996;37(2):263–78. 10.1080/00071669608417858 8773836

[6] Atanasov AG, Waltenberger B, Pferschy-Wenzig EM, Linder T, Wawrosch C, Uhrin P, et al Discovery and resupply of pharmacologically active plant-derived natural products: A review. Biotechnol Adv. 2015;33(8):1582–614. 10.1016/j.biotechadv.2015.08.001 26281720 PMC4748402

[7] Forni C, Facchiano F, Bartoli M, Pieretti S, Facchiano A, D'Arcangelo D, et al Beneficial Role of Phytochemicals on Oxidative Stress and Age-Related Diseases. Biomed Res Int. 2019 Artn 8748253 10.1155/2019/8748253 31080832 PMC6475554

[8] Abarikwu SO, Onuah CL, Singh SK. Plants in the management of male infertility. Andrologia. 2020;52(3). ARTN e13509 10.1111/and.13509 31989693

[9] Sun YL, Tang SB, Shen W, Yin S, Sun QY. Roles of Resveratrol in Improving the Quality of Postovulatory Aging Oocytes In Vitro. Cells-Basel. 2019;8(10). ARTN 1132 10.3390/cells8101132 31547622 PMC6829324

[10] Liu MJ, Sun AG, Zhao SG, Liu H, Ma SY, Li M, et al Resveratrol improves in vitro maturation of oocytes in aged mice and humans. Fertil Steril. 2018;109(5):900–7. 10.1016/j.fertnstert.2018.01.020 29778389

[11] Barbe A, Mellouk N, Rame C, Grandhaye J, Staub C, Venturi E, et al A grape seed extract maternal dietary supplementation in reproductive hens reduces oxidative stress associated to modulation of plasma and tissue adipokines expression and improves viability of offsprings. PLoS One. 2020;15(4):e0231131 10.1371/journal.pone.0231131 . 32282838 PMC7153862

[12] Bagchi D, Bagchi M, Stohs SJ, Das DK, Ray SD, Kuszynski CA, et al Free radicals and grape seed proanthocyanidin extract: importance in human health and disease prevention. Toxicology. 2000;148(2–3):187–97. 10.1016/s0300-483x(00)00210-9 . 10962138

[13] Bagchi D, Swaroop A, Preuss HG, Bagchi M. Free radical scavenging, antioxidant and cancer chemoprevention by grape seed proanthocyanidin: an overview. Mutat Res. 2014;768:69–73. 10.1016/j.mrfmmm.2014.04.004 . 24751946

[14] Olaku OO, Ojukwu MO, Zia FZ, White JD. The Role of Grape Seed Extract in the Treatment of Chemo/Radiotherapy Induced Toxicity: A Systematic Review of Preclinical Studies. Nutr Cancer. 2015;67(5):730–40. 10.1080/01635581.2015.1029639 . 25880972

[15] Downing LE, Ferguson BS, Rodriguez K, Ricketts ML. A grape seed procyanidin extract inhibits HDAC activity leading to increased Pparalpha phosphorylation and target-gene expression. Mol Nutr Food Res. 2017;61(2). 10.1002/mnfr.201600347 . 27624175 PMC5292052

[16] Gonzalez-Abuin N, Martinez-Micaelo N, Margalef M, Blay M, Arola-Arnal A, Muguerza B, et al A grape seed extract increases active glucagon-like peptide-1 levels after an oral glucose load in rats. Food Funct. 2014;5(9):2357–64. 10.1039/c4fo00447g . 25088664

[17] Decorde K, Agne A, Lacan D, Ramos J, Fouret G, Ventura E, et al Preventive effect of a melon extract rich in superoxide scavenging activity on abdominal and liver fat and adipokine imbalance in high-fat-fed hamsters. J Agric Food Chem. 2009;57(14):6461–7. 10.1021/jf900504g . 19601676

[18] Barbe A, Bongrani A, Mellouk N, Estienne A, Kurowska P, Grandhaye J, et al Mechanisms of Adiponectin Action in Fertility: An Overview from Gametogenesis to Gestation in Humans and Animal Models in Normal and Pathological Conditions. Int J Mol Sci. 2019;20(7). 10.3390/ijms20071526 . 30934676 PMC6479753

[19] Liu XT, Lin X, Mi YL, Li J, Zhang CQ. Grape Seed Proanthocyanidin Extract Prevents Ovarian Aging by Inhibiting Oxidative Stress in the Hens. Oxidative Medicine and Cellular Longevity. 2018 Artn 9390810 10.1155/2018/9390810 29541349 PMC5818927

[20] Hou FY, Xiao M, Li J, Cook DW, Zeng WD, Zhang CQ, et al Ameliorative Effect of Grape Seed Proanthocyanidin Extract on Cadmium-Induced Meiosis Inhibition During Oogenesis in Chicken Embryos. Anatomical Record-Advances in Integrative Anatomy and Evolutionary Biology. 2016;299(4):450–60. 10.1002/ar.23320 26799944

[21] Etches RJ, Petitte JN. Reptilian and Avian Follicular Hierarchies—Models for the Study of Ovarian Development. Journal of Experimental Zoology. 1990:112–22. 10.1002/jez.1402560419 1974772

[22] Canepa S BLA, Fagu C, Flon C, Monniaux D. Validation d’une methode immunoenzymatique pour le dosage de la progesterone dans le plasma des ovins et des bovins. Les Cahiers Techniques de L’INRA. 2008;64:19–30.

[23] Mellouk N, Rame C, Delaveau J, Rat C, Marchand M, Mercerand F, et al Food restriction but not fish oil increases fertility in hens: role of RARRES2? Reproduction. 2018;155(4):321–31. 10.1530/REP-17-0678 . 29374087

[24] Pearson AM, Gray JI, Wolzak AM, Horenstein NA. Safety Implications of Oxidized Lipids in Muscle Foods. Food Technology. 1983;37(7):121–9.

[25] Kara K, Guclu BK, Baytok E, Senturk M. Effects of grape pomace supplementation to laying hen diet on performance, egg quality, egg lipid peroxidation and some biochemical parameters. Journal of Applied Animal Research. 2016;44(1):303–10. 10.1080/09712119.2015.1031785

[26] Reis JH, Gebert RR, Barreta M, Boiago MM, Souza CF, Baldissera MD, et al Addition of grape pomace flour in the diet on laying hens in heat stress: Impacts on health and performance as well as the fatty acid profile and total antioxidant capacity in the egg. J Therm Biol. 2019;80:141–9. 10.1016/j.jtherbio.2019.01.003 . 30784478

[27] Galli GM, Da Silva AS, Biazus AH, Reis JH, Boiago MM, Topazio JP, et al Feed addition of curcumin to laying hens showed anticoccidial effect, and improved egg quality and animal health. Res Vet Sci. 2018;118:101–6. 10.1016/j.rvsc.2018.01.022 . 29421478

[28] Dulundu E, Ozel Y, Topaloglu U, Toklu H, Ercan F, Gedik N, et al Grape seed extract reduces oxidative stress and fibrosis in experimental biliary obstruction. J Gastroenterol Hepatol. 2007;22(6):885–92. 10.1111/j.1440-1746.2007.04875.x . 17565645

[29] Choi SK, Zhang XH, Seo JS. Suppression of oxidative stress by grape seed supplementation in rats. Nutr Res Pract. 2012;6(1):3–8. 10.4162/nrp.2012.6.1.3 . 22413034 PMC3296920

[30] Rajput SA, Sun L, Zhang NY, Khalil MM, Ling Z, Chong L, et al Grape Seed Proanthocyanidin Extract Alleviates AflatoxinB(1)-Induced Immunotoxicity and Oxidative Stress via Modulation of NF-kappaB and Nrf2 Signaling Pathways in Broilers. Toxins (Basel). 2019;11(1). 10.3390/toxins11010023 . 30621062 PMC6356337

[31] Bailey RL, Clark GE. Occurrence of twin embryos in the eastern bluebird. PeerJ. 2014;2:e273 10.7717/peerj.273 . 24688852 PMC3961165

[32] Deeming DC. Double-yolked pheasant eggs provide an insight into the control of albumen secretion in bird eggs. Br Poult Sci. 2011;52(1):40–7. 10.1080/00071668.2010.538372 . 21337196

[33] Warren DC S H. The time factor in egg formation. Poultry Science. 1935; 14(4):195–207.

[34] Fasenko GM, Robinson FE, Danforth BL, Zelter I. An examination of fertility, hatchability, embryo mortality, and chick weight in double versus single-yolked broiler breeder eggs. Canadian Journal of Animal Science. 2000;80(3):489–93. 10.4141/A99-090

[35] Jaap RG, Muir FV. Erratic Oviposition and Egg Defects in Broiler-Type Pullets. Poultry Sci. 1968;47(2):417-&. 10.3382/ps.0470417

[36] Fechheimer NS, Jaffe WP. Fertility and embryo death in double-yolked eggs. J Reprod Fertil. 1966;12(2):363–4. 10.1530/jrf.0.0120363 . 5925013

[37] Sahin K, Akdemir F, Orhan C, Tuzcu M, Hayirli A, Sahin N. Effects of dietary resveratrol supplementation on egg production and antioxidant status. Poult Sci. 2010;89(6):1190–8. 10.3382/ps.2010-00635 . 20460666

[38] Xia EQ, Deng GF, Guo YJ, Li HB. Biological activities of polyphenols from grapes. Int J Mol Sci. 2010;11(2):622–46. 10.3390/ijms11020622 . 20386657 PMC2852857

[39] Surai PF, Fisinin V.I., Karadas F. Antioxidant systems in chick embryo development. Part 1. Vitamin E, carotenoids and selenium. Anim Nutr. 2016;31:1–11. 10.1016/j.aninu.2016.01.001 PMC5941026 29767100

[40] Salary J S-A F., Kalantan M., Matin H.R. In ovo injection of vitamin E on post-hatch immunological parameters and broiler chicken performance. Asian Pacific J Trop Biomed. 2014;31:S616–S9.

[41] Hajati H. H A., Golian A., Nassiri-Moghaddam H., Nassiri M.R. The effect of In Ovo injection of grape seed extract and vitamin C on hatchability, antioxidant activity, yolk sac absorption, performance and ileal micro flora of broiler chickens. Res Opin Anim Vet Sci. 2014;4:633–8.

[42] Urso UR, Dahlke F, Maiorka A, Bueno IJ, Schneider AF, Surek D, et al Vitamin E and selenium in broiler breeder diets: Effect on live performance, hatching process, and chick quality. Poult Sci. 2015;94(5):976–83. 10.3382/ps/pev042 . 25713394

[43] Emamverdi M, Zare-Shahneh A, Zhandi M, Zaghari M, Minai-Tehrani D, Khodaei-Motlagh M. An improvement in productive and reproductive performance of aged broiler breeder hens by dietary supplementation of organic selenium. Theriogenology. 2019;126:279–85. 10.1016/j.theriogenology.2018.12.001 . 30594103

[44] Latshaw JD, Ort JF, Diesem CD. The selenium requirements of the hen and effects of a deficiency. Poult Sci. 1977;56(6):1876–81. 10.3382/ps.0561876 . 611492

[45] Gil D, Bulmer E, Celis P, Puerta M. Increased sibling competition does not increase testosterone or corticosterone levels in nestlings of the spotless starling (Sturnus unicolor). Horm Behav. 2008;54(2):238–43. 10.1016/j.yhbeh.2007.11.013 . 18190915

[46] Groothuis TG, Muller W, von Engelhardt N, Carere C, Eising C. Maternal hormones as a tool to adjust offspring phenotype in avian species. Neurosci Biobehav Rev. 2005;29(2):329–52. 10.1016/j.neubiorev.2004.12.002 . 15811503

[47] Meeprom A, Sompong W, Suwannaphet W, Yibchok-anun S, Adisakwattana S. Grape seed extract supplementation prevents high-fructose diet-induced insulin resistance in rats by improving insulin and adiponectin signalling pathways. Br J Nutr. 2011;106(8):1173–81. 10.1017/S0007114511001589 . 21736810

[48] Dupont J, LeRoith D. Insulin and insulin-like growth factor I receptors: similarities and differences in signal transduction. Horm Res. 2001;55 Suppl 2:22–6. 10.1159/000063469 . 11684871

[49] Barbe A, Rame C, Mellouk N, Estienne A, Bongrani A, Brossaud A, et al Effects of Grape Seed Extract and Proanthocyanidin B2 on In Vitro Proliferation, Viability, Steroidogenesis, Oxidative Stress, and Cell Signaling in Human Granulosa Cells. Int J Mol Sci. 2019;20(17). 10.3390/ijms20174215 . 31466336 PMC6747392

[50] Li HX, Chen KL, Wang HY, Tang CB, Xu XL, Zhou GH. Chemerin inhibition of myogenesis and induction of adipogenesis in C2C12 myoblasts. Mol Cell Endocrinol. 2015;414:216–23. 10.1016/j.mce.2015.07.006 . 26164089

[51] Yao J, Li Z, Fu Y, Wu R, Wang Y, Liu C, et al Involvement of obesity-associated upregulation of chemerin/chemokine-like receptor 1 in oxidative stress and apoptosis in ovaries and granulosa cells. Biochem Biophys Res Commun. 2019;510(3):449–55. 10.1016/j.bbrc.2019.01.125 . 30722991

[52] Caimari A, Marine-Casado R, Boque N, Crescenti A, Arola L, Del Bas JM. Maternal intake of grape seed procyanidins during lactation induces insulin resistance and an adiponectin resistance-like phenotype in rat offspring. Sci Rep. 2017;7(1):12573 10.1038/s41598-017-12597-9 . 28974704 PMC5626783

[53] Wang F, Liu Y, Yang W, Yuan J, Mo Z. Adiponectin inhibits NLRP3 inflammasome by modulating the AMPK-ROS pathway. Int J Clin Exp Pathol. 2018;11(7):3338–47. . 31949710 PMC6962845

[54] Lin YT, Chen LK, Jian DY, Hsu TC, Huang WC, Kuan TT, et al Visfatin Promotes Monocyte Adhesion by Upregulating ICAM-1 and VCAM-1 Expression in Endothelial Cells via Activation of p38-PI3K-Akt Signaling and Subsequent ROS Production and IKK/NF-kappaB Activation. Cell Physiol Biochem. 2019;52(6):1398–411. 10.33594/000000098 . 31075190

[55] Oita RC, Ferdinando D, Wilson S, Bunce C, Mazzatti DJ. Visfatin induces oxidative stress in differentiated C2C12 myotubes in an Akt- and MAPK-independent, NFkB-dependent manner. Pflugers Arch. 2010;459(4):619–30. 10.1007/s00424-009-0752-1 . 19898975

[56] Ribas-Latre A, Baselga-Escudero L, Casanova E, Arola-Arnal A, Salvado MJ, Blade C, et al Dietary proanthocyanidins modulate BMAL1 acetylation, Nampt expression and NAD levels in rat liver. Sci Rep. 2015;5:10954 10.1038/srep10954 . 26051626 PMC4603780

