# Supplementary figures and images for: A grape seed extract maternal dietary supplementation improves egg quality and reduces ovarian steroidogenesis without affecting fertility parameters in reproductive hens
Source: PLoS One. 2020 May 14;15(5):e0233169. doi: 10.1371/journal.pone.0233169 (PMC7224513; doi:10.1371/journal.pone.0233169)

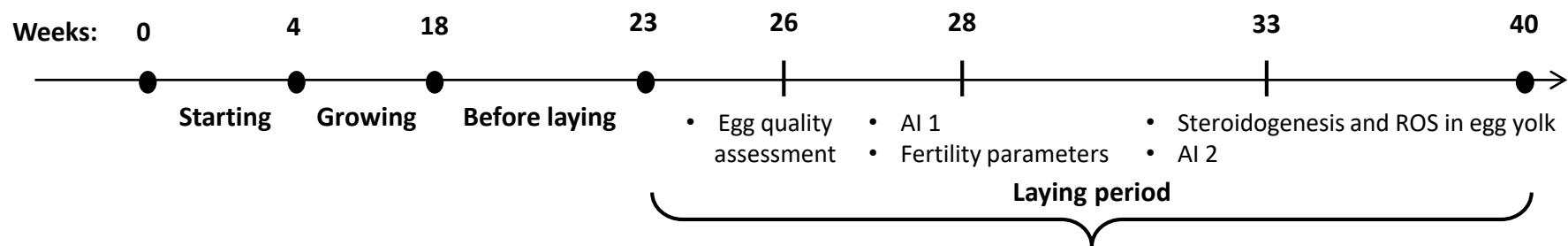

**Experiment 1:**

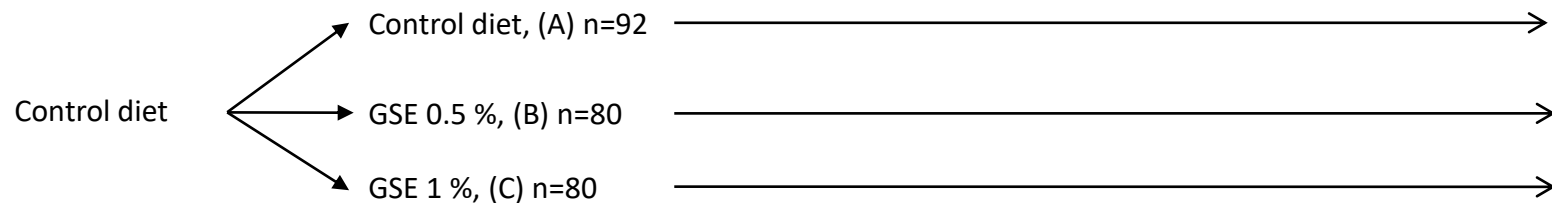

**Experiment 2:**

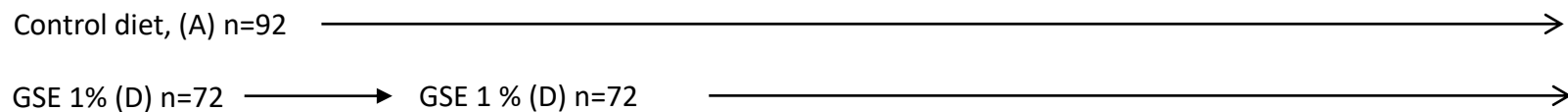

**S1 Fig**

Supplement: S1 Fig — From one to the fourth week of age, 324 female breeder chicks received an ad libitum diet (free access to food), called a starting diet. At week 4, all animals received a restricted diet according to Hendrix Genetics recommendation. From 4 week to 40 week, animals received three different diets: growing (from 4 to 18 week), before laying (from 18 week to 21 week) and laying (from 21 week to 40 week). We performed two experiments according the time of GSE supplementation. In the experiment 1, the animals were divided into three groups: group A (control, n = 92), group B and C supplemented with GSE at 0.5% (n = 80) and 1% (= 80) of the total diet composition, respectively, since the age of 4 weeks until 40 week-old. In the experiment 2, we used two groups of animals: group A (control, n = 92) and group D (supplemented with 1% of GSE since the hatching until 40 week-old, n = 72). For both experiments, eggs were collected during the whole laying period, from 23th to 40th week. At 26th week, the quality of 30 eggs per group was assessed. At 28th week, the first Artificial Insemination (AI1) was performed and the fertility parameters were assessed after the hatching. At 33th week, the steroidogenesis and ROS level in yolk eggs were assessed, for 30 and 10 egg yolks per group of animals, respectively, and then the second Artificial Insemination (AI2) was performed. (PDF) [file pone.0233169.s001.pdf]
