# Supplementary material for: A grape seed extract maternal dietary supplementation improves egg quality and reduces ovarian steroidogenesis without affecting fertility parameters in reproductive hens
Source: PLoS One. 2020 May 14;15(5):e0233169. doi: 10.1371/journal.pone.0233169 (PMC7224513; doi:10.1371/journal.pone.0233169)
Supplement: S1 Table — A: control without supplementation, B and C: supplementation at 0.5% and 1% of the total diet composition, respectively, starting at 4 week-old until 40 week-old (experiment 1), and D: supplementation at 1% of the total diet composition, starting at hatch until 40 week-old (experiment 2). (PDF) [file pone.0233169.s002.pdf]

	Starting		Growing				Before laying				Laying			
**Weeks**	**0 to 4**		**5 to 17**				**18 to 21**				**22 to 40**			
**Diet**	**A**	**D**	**A**	**B**	**C**	**D**	**A**	**B**	**C**	**D**	**A**	**B**	**C**	**D**
Corn	39.64	39.64	36.58	36.58	36.58	36.58	49.33	49.33	49.33	49.33	54.19	54.19	54.19	54.19
Wheat	23.86	23.86	30	30	30	30	22.22	22.22	22.22	22.22	8	8	8	8
Soybean meal	29.3	29.3	3.32	3.32	3.32	3.32	14.37	14.37	14.37	14.37	13.95	13.95	13.95	13.95
Wheat bran	0	0	11.29	11.29	11.29	11.29	0	0	0	0	0	0	0	0
Soy oil	2.2	2.2	1	1	1	1	1.02	1.02	1.02	1.02	2	2	**2**	**2**
Sunflower oil	1	1	14	14	14	14	8	8	8	8	5.8	5.8	5.8	5.8
Sodium Bicarbonate	0.18	0.18	0.1	0.1	0.1	0.1	0.1	0.1	0.1	0.1	0.1	0.1	0.1	0.1
Calcium carbonate	0.86	0.86	1.08	1.08	1.08	1.08	2.1	2.1	2.1	2.1	6.6	6.6	6.6	6.6
Phosphate	2.06	2.06	1.77	1.77	1.77	1.77	2.06	2.06	2.06	2.06	2.2	2.2	2.2	2.2
Salt	0.3	0.3	0.29	0.29	0.29	0.29	0.29	0.29	0.29	0.29	0.28	0.28	0.28	0.28
Methionine DL	0.1	0.1	0	0	0	0	0.01	0.01	0.01	0.01	0.05	0.05	0.05	0.05
Lysine	0	0	0.07	0.07	0.07	0.07	0	0	0	0	0	0	0	0
Mineral premix	0.5	0.5	0.5	0.5	0.5	0.5	0.5	0.5	0.5	0.5	0.5	0.5	0.5	0.5
Soybean	0	0	0	0	0	0	0	0	0	0	6.33	6.33	6.33	6.33
**Grape Seed Extract**	0	1	0	0.5	1	1	0	0.5	1	1	0	0.5	1	1

Table S1: Composition of the diet for the different groups of animals

A: control without supplementation, B and C: supplementation at 0.5% and 1% of the total diet composition, respectively, starting at 4 week-old until 40 week-old, and D: supplementation at 1% of the total diet composition, starting at hatch until 40 week-old.
