# Supplementary material for: A grape seed extract maternal dietary supplementation improves egg quality and reduces ovarian steroidogenesis without affecting fertility parameters in reproductive hens
Source: PLoS One. 2020 May 14;15(5):e0233169. doi: 10.1371/journal.pone.0233169 (PMC7224513; doi:10.1371/journal.pone.0233169)
Supplement: S2 Table — (PDF) [file pone.0233169.s003.pdf]

Table S2: Oligonucleotide primer sequences

Gene	Product size (bp)	Sequence Forward	Sequence Reverse
Actin	188	5'-ACGGAACCACAGTTTATCATC-3'	5'-GTCCCAGTCTTCAACTATACC-3'
GAPDH	198	5'-TGCTGCCCAGAACATCATCC-3'	5'-ATCAGCAGCAGCCTTCACTACC-3'
EIF3F	167	5'-GCACAACGAGTCCGAGGAT-3'	5'-CTTCCCGGCTGTAGTACTCG-3'
RARRES2	314	5'-CGCGTGGTGAAGGATGTG-3'	5'-CGACTGCTCCCTAAAGAGGAACT-3'
CMKLR1	403	5-'CGGTCAACGCCATTTGGT-3'	5-'GGGTAGGAAGATGTTGAAGGAA-3'
CCRL2	391	5'-CACGCAGTGTTTGCTTTAAAAGC-3'	5'-CAACAGCCCACGTGACAATG-3'
GPR1	165	5'-TGTAAAACGCTTTCCCCTTCTCT-3'	5'-ATGTCAGCAACTTCACGCAGA-3'
ADIPOQ	64	5'-ACAGGTGCAGAAGGACCGAG-3'	5-'AAGACAGAGCCGCTTGCTTG-3'
ADIPOR1	350	5'-GAATACACACCGAGACGGGC-3'	5'-GCCCAAGACGCAGACAATGG-3'
ADIPOR2	345	5-'GAGACTGGCAACATCTGGAC-3'	5'-TGCGATGCCCAGGACACAAA-3'
NAMPT	84	5'-CCGATCCAAACAAACGGTCAAAG-3'	5'-TGCCTTCTTCAAGTGTCACATAATC-3'
P450 aromatase	144	5'-TGCTCCTGATACTCTGTCCG-3'	5'-TTGAGGTTTGGCATGTCATC-3'
StAR	102	5'-AAGTGATGGCCCTTATCTCGG-3'	5'-TGGCTGCTACAAACACTGCTG-3'
